# Families adapting to COVID-19 in urban Bangladesh: “It felt like the sky fell apart and we were in shock”

**DOI:** 10.3389/fpsyg.2024.1296083

**Published:** 2024-03-14

**Authors:** Ahmed Jojan Nandonik, Shangjucta Das Pooja, Zarina Nahar Kabir, Shoshannah Kiriam

**Affiliations:** ^1^SAJIDA Foundation, Dhaka, Bangladesh; ^2^Karolinska Institute, Huddinge, Sweden; ^3^School of Allied Health and Human Performance, University of South Australia, Adelaide, SA, Australia

**Keywords:** COVID-19, preventative practices, informal care, lockdown, Bangladesh, health seeking behaviour, mental health

## Abstract

**Background:**

The COVID-19 epidemic has especially impacted the urban population in Bangladesh. Studies on COVID-19 have primarily focused on the patient’s perspective. It is important to understand the experience of family members who adopt caregiving roles, as the experience of COVID-19 also impacts, and is impacted by, household members. This study aimed to explore the challenges, preventative practices, health-seeking behaviour, and perspectives of navigating the health care system from the perspective of family members of persons who had recovered from COVID-19 during its initial outbreak in Bangladesh.

**Methods:**

Participants of this qualitative study were family members (*n* = 7) of persons who had recovered from COVID-19 (either suspected or confirmed). Semi-structured in-depth interviews were conducted over telephone. Thematic analysis was used to analyse the data.

**Results:**

Analysis revealed three key themes: changes in everyday practices and choice of health care, challenges and constraints, and unexpected positive outcomes. All the themes had temporal dimension to them with four distinct phases: early stage of COVID-19, strict lockdown phase, COVID-19 diagnosis and illness period and post COVID-19 recovery.

**Conclusion:**

The importance of maintaining social contact for psychological wellbeing during critical times was evident in the study. Online communication and social media enabled participants to remain ‘socially connected’ which further supported their mental health. Increased attention to hygiene practices both before, during and subsequent to COVID-19 infections within families was reported. Physical distancing in case of a suspected or confirmed COVID-19 case was found logistically and socially impractical in a densely populated city.

## Background

The rapid spread of COVID-19 (SARS-COV-2) caused a global public health emergency commencing in January 2020 (To et al., 2021). Bangladesh is a lower-middle income, densely populated country, with a population of 171 million (The World Bank, 2020).

On the 8th March, 2020, Bangladesh declared its first COVID-19 case, with the government of Bangladesh (GoB) imposing a nationwide lockdown from 26th March, 2020. This lockdown was extended seven times until 30th May, 2020 when restrictions began to be lifted (Khan et al., 2020). All the citizens, except frontline workers were instructed to remain at home with the exception of ‘essential activities’ (grocery shopping and accessing healthcare facilities). Public awareness campaigns regarding COVID-19 preventative practices were disseminated via television, newspapers, and social media.

A government hotline service was established to answer general public COVID-19 related queries. Testing facilities were gradually expanded from 240 tests per million in April 2020 to an average of 5,137 tests per million in the first week of July of the same year (Alif, 2020; Shovon, 2020); and was among the lowest number of tests per million in South Asia (Worldometer, 2021). Initially, the GoB announced that those who were confirmed positive with COVID-19 could receive treatment only in those hospitals dedicated to patients with COVID-19.

The pandemic has disproportionately affected low- and middle-income countries (LMICs) like Bangladesh due to limited resources and pre-existing socioeconomic and health challenges, in contrast to high-income countries (HICs). Evidence shows that COVID-related mortality rates are four times (OXFAM, 2022) higher in poorer countries. While Bangladesh focused on infection control, prolonged lockdowns disrupted daily life, particularly affecting those in informal sectors and labour-intensive industries. Balancing the need for emergency healthcare resources and maintaining economic and developmental activities presented significant challenges for Bangladesh (Ahmed et al., 2023).

Bangladesh is marked by constrained resources, wherein the health system contends with pre-existing challenges (Islam, 2014). Notably, the nation confronts a scarcity of human resources in the health sector, reflected in a doctor-to-population ratio of 0.6 per 1,000 (Pooja et al., 2022). The existing health infrastructure is strained, with a ratio of 7.4 skilled health workers per 10,000 population (Biswas et al., 2020). Furthermore, there is an absence of robust national plans, guidelines, or legislative frameworks necessary for overseeing a global health crisis such as the COVID-19 pandemic. The ramifications of the COVID-19 pandemic have extended beyond the health sector, affecting the overall capacities of both the health and economic systems in Bangladesh (Ahmed et al., 2023).

The urban context was particularly vulnerable to adverse health outcomes during the COVID-19 pandemic (Houweling et al., 2024). Implementing social distancing and rigorous quarantine practices is challenging in urban cities in South Asia due to environmental, behavioural, and economic factors (Kusuma et al., 2021). Urban households in Bangladesh are typically multi-generational families sharing limited space which poses challenges for families in adopting preventative and quarantine practices (Shah et al., 2020). Studies have reported a reluctance among the general public in Bangladesh to engage in preventative practices at the individual level (Ferdous et al., 2020; Hossain et al., 2021), however little is known about the role of families in adopting preventative practices and responding to COVID-19 within the household.

In South Asia, family members have a strong sense of familial care and obligation to ensure the wellbeing of all members within the family unit (Hoole, 2002). Unwell family members largely rely on informal family caregivers, preferring to provide care at home where possible (Alam et al., 2019). While the phenomena of familial and informal caregiving is well documented (Brown and Brown, 2014; Roth et al., 2015), including the changes to pre-existing caregiving roles during the COVID-19 pandemic (Lathabhavan et al., 2023), there is limited literature that focuses on the phenomena of family members who became caregivers as a result of the COVID-19 pandemic in low and middle-income country contexts (Mackworth-Young et al., 2020; Rizvi Jafree et al., 2020). Health care decisions are typically made by family members and relatives and health care providers on behalf of an unwell family member, particularly children and the elderly (Russel, 2004). There is a need to explore the knowledge, everyday practices, and health-seeking practises of family members who have cared for a person with COVID-19, given their central role in care and caregiving in this context. The aim of this study was to explore the experiences of challenges, preventative practices, health-seeking behaviour, and navigating the health care system from the perspective of family members of persons who had recovered from COVID-19 during the initial outbreak in Bangladesh.

## Methods

An exploratory descriptive qualitative study design was employed to gain an in-depth understanding experiences of families, in recognition that there was limited existing data about this emerging phenomena (Kim et al., 2017). Participants were family members of persons who had recovered from COVID-19 (either suspected or confirmed) and received treatment either at home, in a hospital or isolation centre. The researchers worked with a local organisation, SAJIDA Foundation to recruit participants.

SAJIDA Foundation turned their non-profit hospital in Narayanganj (27 km from Dhaka) into a treatment facility dedicated to patients of COVID-19 and established a telehealth hotline to provide free medical advice to the public during the pandemic. Participants of the current study were purposively selected by doctors who had provided services, either via the hotline or in the hospital. Doctors were briefed with the inclusion and exclusion criteria, including someone whose family member (who also happens to be a household member) had specific symptoms of COVID-19 and either was recovered and discharged from the hospital or completed the 14-day isolation period at home. Researchers telephoned the identified participants, and asked if any family member in their household would be willing to engage in an interview. Family members experiencing any critical illness and who did not have access to mobile phones were excluded. Ten family members were approached for an interview, of whom three declined, resulting in seven study participants. Amid the initial stages of the COVID-19 pandemic, heightened stress and lockdowns involving future uncertainties created a complex situation for potential participants to participate in an interview. Approaching a limited number of participants reflected a recognition of these exceptional circumstances and balancing research goals with ethical considerations. Five participants were women and two men. Participants were either a spouse (*n* = 4), child (*n* = 2) or a daughter-in-law (*n* = 1) to the person with suspected or confirmed COVID-19 case.

Semi-structured interviews were conducted via telephone to comply with pandemic restrictions. The two interviewing researchers both held undergraduate degrees, one in the health field. Their research training and supervision was conducted by the other members of the research team who were experienced researchers who held post-graduate qualifications, and had extensive health and context specific experience. Each interview lasted for 40–60 min and was audio recorded. Informed consent was obtained before the commencement of the interview. Participants were informed of the study’s objectives, how their data would be stored and used, that participation was voluntary and that he/she could refuse to answer any question and could withdraw from the study at any point, even after completion of the interview. Researchers de-identified all the participants by assigning each of them a unique identification number to ensure anonymity. All data is stored in a password-protected cloud which can be accessed only by the researchers. The interview was conducted through Microsoft Teams, and the system recorded audio. ID numbers were assigned to ensure anonymity while the data was being transcribed.

All interviews were transcribed verbatim in Bangla and then thematically analysed (Braun and Clarke, 2012). The co-authors read the transcripts multiple times (all transcripts by AJN and SDP, three by ZNK and transcripts translated in English were read by SK) to familiarise themselves with the data. Three transcripts were selected for all authors to code. These codes were then compared and an agreed upon initial coding frame was collaboratively developed to ensure inter-coder reliability, minimise bias, and ensure rigor. If new codes emerged during in-depth analysis, the coding frame was adapted following discussion within the team. Once initial coding was completed, the team collaboratively identified categories and broad themes which were developed and consolidated on discussion.

Direct quotes were used while describing a participant’s experience to ensure that the findings accurately reflected participants’ experiences. Quotes that described similar phenomena by multiple participants were considered when selecting quotes. Key quotes presented in the paper were then translated into English and compared back to the Bangla transcripts. Quotes by specific participants are referred to as “P” and the participant’s number.

This study was conducted under the purview of SAJIDA Foundation which did not require a formal ethical approval, particularly due to the non-invasive nature of the study. Ethical guidelines outlined in the Declaration of Helsinki was followed in the study. No institutional ethical approval was required.

### Rigor and trustworthiness

Several steps were taken to promote the credibility, dependability, confirmability, and transferability of this study (Liamputtong, 2010). Researchers initially immersed themselves in the caregiving literature and emerging studies around COVID-19 to understand the contextual phenomena of the pandemic and enhance their sensitivity during interviews. The interview guideline was prepared by the research team to reflect the key research aims. The guideline was piloted with two participants who met the selection criteria by the two interviewing researchers and then subsequently refined by the research team to ensure the appropriateness of the guideline. The two interviewers maintained a diary and brainstorming toolkit that documented emerging themes and insights following interviews, which later was reviewed by all collaborating authors through multiple debriefing sessions (Liamputtong, 2010). Peer debriefing after the final interview revealed that there was no new themes emerging during data collection (Fusch and Ness, 2015). Hence, the sample size was deemed adequate by the authors (Vasileiou et al., 2018).

## Results

Analysis revealed three key themes: changes in everyday practices and choice of health care, challenges and constraints, and unexpected positive outcomes. All the themes had a temporal dimension to them, with four distinct phases emerging throughout the experiences of the family members. The ‘early stage of COVID-19’ is defined as February—early March 2020, when there was no restriction from the authority regarding public movement. The ‘strict lockdown phase’ lasted from March 26 until the end of May 2020. The ‘COVID-19 diagnosis and illness period’ is defined as the period when one of the family members of the participants started to exhibit symptoms of COVID-19 and lasted till he/she was recovered. The final ‘post COVID-19 recovery’ phase is defined as when the family member had recovered from COVID-19.

### Changes in everyday practices and choice of healthcare

Almost all participants discussed the significant changes in their day-to-day practices starting from the early stage to post COVID-19 recovery phase.

Early stage of COVID-19: During this period, participants discussed being unsure of the implications COVID-19 held for Bangladesh. Initially, there was little impact of the impending COVID-19 pandemic on everyday practices due to the role of media in perpetuating the perception that COVID-19 would not ‘come to or spread in’ Bangladesh, and no official restriction on public movement. There was a gradual shift in media coverage that highlighted the severity of the pandemic, however due to uncertainty of the authenticity and reliability of media coverage, this had a limited impact on everyday practices. Participants discussed misinformation, which confused people or lulled them into a false sense of security. As P4 highlighted.

“… it (virus) will not survive in temperature in Bangladesh. So, I took it (the situation) very casually.”

Participants discussed experiencing fear and panic once COVID-19 was reported as spreading within Bangladesh. Four out of seven participants expressed fear, P5 recalled.

“I had a feeling that COVID was not there, but when it slowly started to affect the whole world…. when it started in Bangladesh, I was convinced that it was really going to be an awful thing.”

Most participants reported that the guidance on preventative practices obtained from different sources like TV, print media, social media and internet browsing had been generally helpful, although some stated that these only became clearer later during the pandemic.

Strict lockdown phase: The government imposed a nationwide ‘lockdown’ from 26th March, 2020. All participants reported making changes to their everyday practices and developed alternative solutions to adhere to the new restrictions. All seven participants adhered to lockdown and made adjustments, P5 reported.

“We were not that serious at the beginning of the lockdown. But … when everyone understood how serious it was, everything was shut down. Everyone was obeying the restrictions … Then we [the household] got serious about it … let us stay at home and let us not go out.”

Risk calculations were conducted by households to determine changes in everyday practices. Activities ‘outside’ the home were regarded as ‘higher risk’ and the dilemma of when to ‘go outside’ was a topic of much conversation. Typically, grocery shopping and employment were considered essential activities with families adapting these tasks by either shopping in bulk or online whenever possible. Other activities that were considered essential included employment (especially frontline workers) as well as attending religious rituals such as a close relative’s funeral. P2 explained grocery shopping as a key essential activity.

“I have not stepped outside of the house for two months … Only my husband went out shopping….”

Deciding who would be the person ‘going outside’ also became a key household decision, with typically a young, healthy male family member taking this responsibility.

Practices were adopted to prevent COVID-19 from ‘entering the home’, with strategies varying between families.

P6 explained.

“[The residents of our building] took a few initiatives for the building, like domestic help and workers from outside …. we stopped their entry to the building… After coming (from outside), [we] take a bath, keep clothes separated, and then wash them. Separating and washing grocery items. The items that could not be washed were put out in the sunlight.”

P7 noted that some practices, such as wearing a mask constantly, were “annoying.” P7 expressed.

“It is a little annoying to wear masks, sometimes it is difficult to breathe, and it is always terrifying. I mean, should I touch it (to adjust) or sanitise my hand first? I’m sweating, sweat is coming into my eyes but still refraining myself from touching the mask.”

Wearing masks was viewed as an essential preventative and socially accepted practice by all participants’ families during the lockdown period, despite the challenges.

COVID-19 diagnosis and illness period: This was the period that commenced when a member of the household first exhibited symptoms and caused most abrupt and noticeable changes in everyday practices. Participants discussed the concept of isolation, which was described as the family member who was suspected to have contracted COVID-19 staying in a separate room. P1 and P5 talked about arranging a separate room when one of the family members started showing COVID-19 symptoms. P5 stated.

“…when my husband felt body ache and fever…he needed to be isolated in a separate room … I used to leave his meal outside his door, used to wear mask … he said to everyone ‘do not come near the room, especially father, mother, aunt they should not come.’ He added ‘only you can come to provide daily meal and other things that I need but do not enter the room. Put things outside. I’ll take them myself, no need for you to wait’.”

Maintaining social contact with the household member in isolation was considered essential by the families. Participants specified that whilst the family member with COVID-19 (suspected or confirmed) was isolated physically, they still remained socially connected. Participants talked about adopting different strategies to maintain both the physical and mental wellbeing of the ill member, without compromising their safety. P5 used to connect with her husband via online video calls to enable her children to talk with him without putting themselves at a health risk. P1 spoke with passion.

“Yes, it is instructed to keep the distance which I agree with, but every family should chat and spend time with the person, take care of the person, make sure that s/he has taken medicine.”

Maintaining social contact ensured mental wellbeing of all family members and was described as giving them the strength to cope during this stressful period.

Participants discussed that they preferred the home as a place of isolation and recovery, and that they did not want their patient to get admitted to the hospital. They expressed their concern about the unhygienic condition at the public hospitals and the inadequacy of hospital facilities. Four out of seven participants preferred isolation at home over hospital, P7 elaborated.

“We could guess from the very beginning that there were not enough places at hospitals for admission in our country. In our district, there is only one hospital. I got to know from others that the environment there was not hygienic. We thought this could cause even more spread of the infection, so we preferred treatment at home. After continuing medicine, it seemed that (grandmother) was healing, so we did not think about taking (her) to any hospital.”

When in need of a healthcare professional’s advice, participants mentioned that they were more comfortable contacting a doctor who is a close relative or a family friend over the phone. Others mentioned calling hotline service to seek professional advice.

Post COVID-19 recovery phase: Even after the family member with COVID-19 was declared as recovered by a physician, most participants mentioned sustained behavioural change in hygiene practices during the post recovery period. Participants believed that practising good hygiene has a long-term impact on health and would protect from future viral or bacterial diseases. P1 mentioned.

“If these habits are always maintained, then we will not only be protected from COVID-19 but also from viral or bacterial diseases.”

P6 expanded.

“There has been a change, be it for fear of Corona, [but] the end result has been good. …cleanliness is being maintained more than before. Earlier, people used to throw garbage anywhere. Now they use specific places, be it a preventive measure or fear of being scolded by someone. Hence, this is good in terms of our (residential) building.”

However, behaviour change was not always sustained within the public sphere, which was concerning to participants. P3 explained.

“I feel that people have become less conscious (about preventative practices) than before. Like, previously many used to wear masks, but now not many do so.”

Even participants families became less careful over time, according to P4.

“Later on, when the lockdown was relaxed…we also became a little careless. We thought we could go out.”

Participants’ tendency to go back to the pre-COVID-19 lifestyle indicates that even though people had developed awareness about preventative practices over the period of the pandemic, the sustainability of these practices were not always practical.

COVID-19 significantly impacted everyday practices for families and caregivers. The COVID-19 diagnosis and illness period led to substantial changes for families, necessitating changes in hygiene, social practices and use of often limited space within homes to ensure safety among family members. Some of these changes continued throughout the post-COVID-19 recovery phase. Preferences to use hotlines/virtual care services over in-person visit to a hospital were generally preferred.

### Challenges and constraints

The spread of COVID-19 in Bangladesh exposed participants to social, economic, and psychological challenges. Participants indicated that these challenges emerged mostly during the strict lockdown and during the illness phase.

Strict lockdown phase: Families faced financial difficulties during the lockdown as they were unable to engage in paid employment due to mobility restrictions. Some drew on their savings, while others took loans to meet expenses. Three participants reported financial struggle, P1 discussed.

“Gradually,…lower-middle-class families like ours were facing a financial crisis. In this way, the lockdown seemed harmful to us.”

In addition, the pandemic drove up the prices of essential goods making it difficult for participants to afford items such as food and hygiene materials. P3 highlighted the sudden rise in price.

“… during the time of lockdown, the price of masks rose drastically, and it was tough for a middle-class family to afford. It can be said that it became ‘shonar horin’.

‘Shonar horin’ literally translates as ‘a golden deer’ or ‘hard to find.’ Participants also expressed their boredom and uneasiness of continuously staying at home. P4 spoke of the monotony of staying home all the time.

“I felt a little abnormal when everyone was sitting at home as we usually could not stay at home at all. I did not like it and I wanted to go out.”

The financial uncertainty and monotony caused significant pragmatic challenges and mental stress for families.

COVID-19 diagnosis and illness period: P2 shared the immediate reaction of her family when they heard that her husband was tested positive for COVID-19.

“At first, when we came to know that he (participant’s husband) got affected by coronavirus, I would say that it was like the sky was falling apart and we were in shock. I could not believe it and did not know what to do. My husband started crying, thought that he would not survive. He started calling his relatives in the village and cried.”

Maintaining complete physical isolation was challenging, if not impossible for several families. Due to limited space, they were unable to maintain recommended isolation. P7 discussed that they were not “mentally prepared” to keep distance within the household when the spouse was exhibiting symptoms. P7 explained.

“Three of us started to use three different bathrooms. [We] stayed in separate rooms…. [but] we used to eat together, [it] seemed like we were not always keeping distance.”

In addition, most of the participants commented on the challenges of accessing and obtaining a COVID-19 test and the limitations of health care provided in hospitals. Participants were unsure of what to do next, due to the delay in getting test results for COVID-19. Two of the participants P5 and P3 shared their experiences.

“[My husbands] symptoms were starting to appear one by one… he thought of doing COVID-19 test. It was the time when getting for an appointment for a COVID-19 test was difficult. Two days later, he went to *** hospital and had to stand in queue for a long time. He came back as he could not do the test. Later on, he did his test from another hospital on the same day, and one of his friends helped him get the appointment for the test.” (P5).

“When we did the test for the first time, the report came too late. Honestly, when it comes to our health system, a lot of things are not in place. My grandmother’s report came after her death.” (P3).

Participants also discussed the challenges of obtaining admission for those suspected to have COVID-19 or had tested positive. During early 2020, hospitals in Bangladesh were either ‘COVID designated’ (receiving patients who had a confirmed test result) or ‘Non-Covid’ hospitals. ‘Non-Covid hospitals’ routinely turned away persons exhibiting symptoms of COVID-19, on the basis of preventing the spread of the infection within their hospital. P6 faced the challenging situation of a ‘Non-Covid hospital’ refusing admission to his father as he was exhibiting symptoms of COVID-19, whilst a ‘COVID designated’ hospital also denied admission due to the absence of a positive test report. The father’s condition had deteriorated significantly by the time he received the test result. P6 stated that.

“It took three days for the result to come and by the time his (father) condition gradually deteriorated.”

The final key challenge was the stigma associated with a positive COVID-19 test. Most participants said that they faced harsh behaviour from their neighbours and the local community when a member of the family had tested positive. Participants spoke of getting an eviction notice from the landlord. Some felt helpless when their residential building was marked as restricted zone, and no one was allowed to enter or go out. P3 expressed his frustration,

“… the society portrayed us as someone contemptible. Everyone had a negative perception about us saying ‘they have corona at home’. We do not have any relatives here in Dhaka… so we did not have anyone who could offer a helping hand. The government-imposed lockdown and our house was under lockdown for 24 days, but no one came to enquire about us.”

P2 discussed not only facing discriminatory behaviour during the illness period but even after recovery,

“The challenges were mainly social. Our landlord told us to leave and go back to our village. People looked down on us. We faced problems even after our 14-day quarantine period.”

The challenges of stigma, difficulties of physical isolation due to limited space at home, navigation and inefficiency of the health systems faced by the participants was most acute during the illness period of their family member. Financial struggles and the monotony during the strict lockdown phase also contributed to the mental stress of participants.

During the strict lockdown and illness phases, families faced multiple social, psychological, economic and health system related challenges. These included being cut off from social connections, boredom, uncertainty about the future, loss of work and income, as well as stigma associated with diagnosis. Challenges in navigating the health system and obtaining admission to health care services were also part of the experience.

### Unexpected positive outcomes

Despite the challenges and stressors associated with lockdown and family members being ill, participants talked about some unexpected positive outcomes of the pandemic. These were predominantly experienced during strict lockdown and the illness phase.

Strict lockdown phase: Participants mentioned finding relief and enjoyment because they were able to spend much more quality time with their families. P4 stated.

“…actually, everyone is busy here in Dhaka. Due to this lockdown, family attachment has developed. Amidst everyone’s busy times we got to spend time together, this has developed.”

Participants described the pre-COVID-19 period as ‘busy time’, which did not give their family the opportunity to spend quality time together. Lockdown was an opportunity for families to strengthen their relationships by playing indoor games with family members and spending time with each other.

COVID-19 diagnosis and illness period: Some participants stated that during the illness period of their family members they received help and mental support from their relatives, colleagues, neighbours and friends, which helped them which helped them to cope with this challenging period. Four out of the seven participants were grateful of the support they received, as P5 elaborated.

“…when my relatives came to know (about husband being positive for COVID-19) everyone was really supportive. We were blessed by Allah; everyone was continuously taking our updates over phone, including our relatives. No one was negative towards us. Everyone always spoke positively …it was really very important to receive that mental support. They (neighbours and relatives) used to give us whatever we needed.”

Others also spoke of realising the importance of support for mental strength as crucial in the COVID-19 healing process.

Spending quality time within family members and receiving help and support from social networks were unexpected and positive outcomes of the COVID-19 pandemic. Practical and mental support from different quarters was crucial during the recovery process of their family members.

## Discussion

This study explored the experiences of family caregivers to persons who had recovered from COVID-19 and focused on their concerns and challenges regarding preventative practices, the role of information sources, and the experience of navigating the healthcare system. Analyses of the data resulted in three key themes: Changes in everyday practices and health-seeking behaviour, challenges and constraints, and unexpected positive outcomes through the different phases of restrictions during the early phase of the COVID-19 pandemic in Bangladesh.

People faced difficulties both logistically and socially in maintaining social distance in the context of Bangladesh (Shammi et al., 2020). Housing in urban areas is expensive and typically crowded given the countries high population density and rate of urbanisation (World Data Bank, 2018; UNDP Bangladesh, 2019). The social norm of multi-generational living in limited space made it difficult, even impossible, for families to maintain the recommended social distancing, wellbeing and isolation practices (Anwar et al., 2020). These practical challenges for families may have more immediately undermined their self-efficacy, with implications for mental health (Diotaiuti et al., 2021; To et al., 2021). As such, participants took time to adjust to the ‘new normal’.

Ensuring social distancing whilst maintaining social connectedness was crucial for participants. ‘Social distancing’, a concept promoted by the World Health Organisation (WHO, 2020), is a misnomer for what is meant to be ‘physical distancing’. In fact, this study highlights the importance of maintaining social connectedness for the patients’ and the family members’ mental wellbeing. Social support and empathy are crucial in assisting individuals to cope with psychological stress during illness (Mak et al., 2009; da Cruz et al., 2022). Conversely, Williams et al. (2020) highlighted that those who maintained social distance and decreased social interaction during the pandemic reported negative impacts on mental health and wellbeing (Andrade et al., 2023). Social support and empathy are crucial in assisting individuals to cope with psychological stress during illness (Mak et al., 2009). Regular interaction between family members and their relatives via mobile phone or online video chat, as reported in this study, reduced stress for both the ill persons and the family members caring for them.

Indeed, the government-imposed lockdown and restrictions on mobility had the unexpected benefit of bringing many families closer. This time enabled families to spend longer periods of quality time with each other, which had not been previously possible due to competing obligations outside the home, long hours at work, and time wasted in city traffic. As such, lockdowns created some unexpected positive experiences (Rizvi Jafree et al., 2020).

Job and wage loss during lockdowns took a significant financial and emotional toll on families. This was not unique to Bangladesh, with participants from another study finding that the loss of income and disruption in daily routines further contributing to mental wellbeing in the United Kingdom (Williams et al., 2020). Given that Bangladesh is a lower-middle income country, job security, wages and savings are typically low, and social safety nets offered by the government are negligible. Whilst the government, humanitarian organisations and private sector provided some food relief during the ‘lockdown’, the support was focused on the poor who support families with their daily income (e.g., beggars, day-labourers transport workers and roadside entrepreneurs), and those employed in specific prioritised sectors (e.g., export oriented sectors, small and medium enterprises; Foyez, 2020; Mamun, 2020). Limited assistance was available to low-income and middle-income families. As such, an additional 36.9 million (22% of the total population) of the population became ‘new poor’ during 2020, which is attributed to the economic impact of lockdowns on lower-middle income families (Rahman (2020)). A lack of financial security was a major threat to the participants’ subsistence and psychological wellbeing which led them to defy lockdown and find ways to mitigate financial uncertainty.

Participants gradually adapted to the new lifestyle around the pandemic of COVID-19. In order to maintain preventative measures, families adopted alternative ways to meet their daily needs, such as shopping online and interacting socially online instead of venturing out, thus avoiding exposure. Online communication during the restrictions appeared to be the safest and most reliable medium to stay socially connected. Online shopping for groceries boomed in the capital city during the period both in terms of demand and supply of services.

Stigma, discrimination and negative emotions like fear, anxiety and helplessness towards persons with an infectious disease during a pandemic have been reported in earlier studies (Bohle, 2013; Shultz et al., 2015; Choi, 2021). Fear and anxiety were particularly pronounced for participants with older persons at home and with pre-existing chronic conditions (Takashima et al., 2020). The current study participants expressed similar fear of seeing the rising number of deaths during the pandemic. The uncertainty and lack of knowledge about COVID-19 contributed to this fear. Social anxiety can trigger social stigma leading to discriminatory behaviour towards the ill as it was observed when little was known about transmission and treatment of other infectious diseases (Bohle, 2013). Similarly, in this study discriminatory behaviour towards family members of patients with COVID-19 was reported, with families asked to leave their homes or refused any kind of support.

Participants of the current study reported being more attentive to hygiene practices compared to the pre-COVID-19 period. This was attributed to continuous media focus on behavioural changes related to COVID-19 and a central topic of discussion among peers. Participants mentioned being more vigilant outdoors than indoors at home regarding washing hands as they saw the home as a safe sanctuary. Participants in our study seemed concerned about not bringing coronavirus from outside, hence disinfecting everything brought from outside and treating the virus as ‘dirt’. They appeared less concerned about the significant threat of contracting COVID-19 inside one’s home. Previous studies on the secondary transmission of COVID-19 indicate if a member of the household contracts COVID-19, it could easily spread to other members (Nishiura et al., 2020; Shah et al., 2020). Surface transmission of COVID-19 is not as significant a threat as it was thought to be (Davey, 2021), highlighting the key role of airborne transmission within poorly ventilated spaces (such as a home or workplace setting).

Given limited resources, inadequacies in health care services during the pandemic were inevitable (Al-Zaman, 2020; Khan et al., 2020). Participants showed lack of trust and reliance in the healthcare system to tackle the spread of COVID-19. Initial lack of efficiency in obtaining test results (Cousins, 2020) and hospitals turning away patients, indicated a poor responsiveness of the healthcare system. Consequently, home-based management of COVID-19 was preferred over seeking treatment at hospitals. Future preparedness for pandemics is crucial to ensure systems are in place to address similar emergencies, with consistent, accessible and targeted public health messaging to support caregivers navigation of the health care system. Given the preference to care for family members at home, the digitalisation of healthcare services, including investment in hotline or video services may further support improved access to health care services (Chowdhury et al., 2021).

Family caregiving has emerged as a crucial and primary support system, particularly in Low- and Middle-Income Countries (LMICs; Oyegbile and Sibiya, 2022). The study underscores the significant role played by family members in providing initial assistance to COVID-19 patients, a support system that continues to be prevalent throughout the recovery phase. The reliance on family caregiving raises important considerations for future pandemics, necessitating culturally and geographically tailored interventions to address these societies’ unique challenges and strengths.

The study particularly highlighted the difficulty of maintaining social distancing measures in densely populated urban settings within LMICs. The research provides a nuanced understanding of how living conditions impact individuals’ ability to adhere to preventive measures. Densely populated areas not only pose logistical challenges for maintaining physical distance but also shed light on the socio-economic factors that contribute to the spread of infectious diseases (Sahasranaman and Jensen, 2021). This insight emphasises the importance of policy makers and public health messaging to provide advise and recommendations that are context-specific. The diverse conditions and challenges faced by individuals in LMICs, including logistical, practical and financial constraints to adhering to public health measures must be considered when providing advice to ensure adhering to advice is feasible and realistic. It is also recommended that health services be equipped with practical and logistical support of families with COVID-19 or other transmissible diseases that require isolation support.

While much attention has been devoted to the negative consequences of lockdowns, the study also highlights a less-explored dimension—the positive impact on family dynamics (Mercier et al., 2021). Contrary to the predominant narrative of increased stress and strain on relationships during lockdowns (Mari et al., 2020), the research identifies unexpected positive experiences, such as heightened family closeness. This challenges the oversimplified view of lockdowns solely as disruptive forces and underscores the need to recognize the multifaceted nature of their impact on individuals and families. Acknowledging both the challenges and the positive aspects of lockdowns is crucial for developing comprehensive strategies that consider the holistic well-being of families and communities. It is recommended that future research further explores the stressors and protective factors for family caregivers’ mental health in Bangladesh when providing care at home.

One of the challenges when conducting this study was the online nature of data collection due to pandemic restrictions. Researchers worked to establish rapport, asked if participants were alone in the room to ensure confidentiality and checked that participants were comfortable before engaging in in-depth questions within interviews. Limitations of the study include the small sample size due to challenges with recruitment and timing of data collection. The study also only focused on the single setting of the experiences of those living in one city. Inclusion of family members caring for a person with COVID-19 in other urban and rural areas would have enriched the study.

## Conclusion

Family caregivers adapted their health behaviour through improved health and hygiene practices during the pandemic. The importance of maintaining social contact for health and wellbeing was a key theme. Online communication and social media helped participants and their families to remain ‘socially connected’ instead of ‘social distancing’, which helped support wellbeing and the healing process. The recommendation of physical distancing in case of suspected or confirmed case of COVID-19 was found logistically and socially impractical in densely populated communities such as urban Bangladesh.

## Data availability statement

The raw data supporting the conclusions of this article will be made available by the authors, without undue reservation.

## Ethics statement

Ethical approval was not required for the studies involving humans because of the non-invasive nature of the study. However, ethical guidelines as outlined in the Declaration of Helsinki were followed in the study. The studies were conducted in accordance with the local legislation and institutional requirements. Written informed consent for participation was not required from the participants or the participants’ legal guardians/next of kin because recorded verbal informed consent was obtained from the study participants.

## Author contributions

AN: Conceptualization, Data curation, Formal analysis, Investigation, Methodology, Writing – original draft. SP: Conceptualization, Data curation, Formal analysis, Investigation, Methodology, Writing – original draft. ZK: Conceptualization, Data curation, Formal analysis, Methodology, Supervision, Writing – original draft, Writing – review & editing. SK: Conceptualization, Data curation, Formal analysis, Investigation, Methodology, Supervision, Writing – original draft, Writing – review & editing.
